# Facilitating co-research: lessons learned from reflection forms within three participatory action research projects

**DOI:** 10.1186/s12961-024-01210-x

**Published:** 2024-08-23

**Authors:** Helga Emke, Ann Vandendriessche, Mai Chinapaw, Benedicte Deforche, Maïté Verloigne, Teatske Altenburg, Manou Anselma

**Affiliations:** 1 Department of Health Sciences, Faculty of Science, Vrije Universiteit Amsterdam, Amsterdam, The Netherlands; 2 Department of Public Health and Primary Care, Faculty of Medicine and Health Sciences, Ghent University, Ghent, Belgium; 3 Department of Public and Occupational Health, Amsterdam University Medical Centre, Vrije Universiteit Amsterdam, Amsterdam, The Netherlands; 4 Movement and Nutrition for Health and Performance Research Group, Faculty of Physical Education and Physical Therapy, Vrije Universiteit Brussel, Brussels, Belgium; 5 Mulier Instituut, Utrecht, The Netherlands; 6 Health Behaviour and Chronic Diseases and Methodology, Amsterdam Public Health Research Institute, Amsterdam, Netherlands

**Keywords:** Facilitation, Co-research, Participatory action research, Reflection

## Abstract

**Background:**

Mutual learning and shared decision-making are key elements of Participatory Action Research (PAR), highlighting the important role of the facilitator to support this. This study aims to illustrate how a facilitator can contribute to successful PAR sessions based on the reflection of three PAR projects.

**Methods:**

Participatory sessions took place with adolescents for 3–4 school years. After each session (*n* = 252 sessions across three projects), facilitators filled in a reflection form that assessed the group process and their facilitating role. Facilitators independently coded a selection of 135 reflection forms partly deductive and partly inductive based on core PAR principles derived from a pragmatic literature search.

**Results:**

A well-prepared session – for example, including active and creative participatory methods and a clearly stated goal – contributed to efficiency and the necessary flexibility. Making agreements, making sure everyone is heard and taking 'fun-time' appeared important for creating and maintaining a safe, functional and positive atmosphere. Finally, facilitators needed to encourage co-researchers to take the lead and adapt to the group dynamics, to ensure ownership and shared decision-making.

**Conclusion:**

In-depth qualitative analyses of a standardized reflection form used in three different PAR projects resulted in various lessons to support facilitators in collaborating with co-researchers in PAR projects.

**Supplementary Information:**

The online version contains supplementary material available at 10.1186/s12961-024-01210-x.

## Highlights

- Detailed preparation of participatory sessions with multiple scenarios allows the flexibility needed for productive sessions.
- Ensuring a positive, safe and functional atmosphere encouraged productive participatory sessions.
- Facilitators need to continuously adapt to the characteristics, moods and emotions of co-researchers.
- We present an improved reflection form to support facilitators in optimizing their PAR sessions.

**Supplementary Information:**

The online version contains supplementary material available at 10.1186/s12961-024-01210-x.

## Introduction

Participatory action research (PAR) is a promising approach to improve health and reduce health inequities. In this approach, there is collaboration and shared decision-making between researchers and the population of interest to develop actions improving that population’s own situation [1]. Actions developed using PAR are more likely to meet the needs and preferences of the population of interest, and thereby may be more effective than traditional more top-down developed actions [2]. Mutual learning and respect are essential in the participatory process, with participants’ experiences valued as a legitimate form of knowledge that can influence practice [1]. In the participatory process, participants are trained as co-researchers to provide them with knowledge, skills and abilities to conduct research in their own particular context and that of their peers [1, 3]. Particularly for children and adolescents, participating in PAR and, thus, being a co-researcher can improve their individual development, empowerment and critical awareness of societal issues [4].

When conducting PAR, there are several core principles to take into account [5]. Wright and colleagues identified 11 common participatory principles, including that co-research should promote critical reflexivity of co-researchers and academic researchers [6]. Academic researchers will become better researchers when they reflect on their behaviour, thoughts and co-operation during their collaboration with co-researchers [7]. This is especially important when academic researchers act as facilitators of the PAR process, for example, by reflecting on power differences between the academic researchers and the co-researchers [5]. Reflexivity reveals the influence of the facilitator on the PAR process, the generated data and the group dynamics [8]. However, guidance on how to structurally reflect on and improve the role of the facilitator is currently lacking [9].

Due to the grant-based funding of academic research, in most projects the overall aim and research questions are already set. Within these boundaries, researchers can and should still promote a shared and dynamic PAR process, endorsing mutual learning and decision-making, where co-researchers can contribute to the best of their potential [9]. Facilitators who are flexible and open-minded and who have good verbal and written communication skills and the ability to maintain a supportive and encouraging attitude are generally capable to create such a PAR process [10]. However, apart from more general guidelines [5], there is currently a lack of data of how PAR can be successfully facilitated. Critical reflection by facilitators can provide insight in success factors of a good PAR session. This insight can further improve future PAR projects.

The current study aims to illustrate how a facilitator can contribute to a successful PAR process based on standardized reflection forms collected in three PAR projects with children and adolescents. This paper presents how a standardized reflection form can inform the facilitators in improving their PAR facilitation in practice, including specific examples, lessons learned and recommendations from the three PAR projects using this form. Based on the analysis of the reflection forms, suggestions will be made to improve the reflection form.

## Methods

In the following sections, we give a description of each project, the design of the participatory sessions across the three projects, the reflection form and how the forms were coded and analysed based on a pragmatic literature research.

### Projects

All three projects focused on improving one or multiple energy balance-related behaviours in children and/or adolescents. The leading academic researchers (H.E., A.V., M.A.) collaborated with their co-researchers in so-called Action Teams, consisting of 3–12 children or adolescents. The academic researcher had a facilitating role. Often a second facilitator (e.g. an intern) was present to assist in the process. We obtained a written active informed consent to participate in the participatory process from at least one of the parents or guardians in all three projects and in the LIKE and Healthy sleep project also from the co-researchers themselves. Table 1 presents more information on the three PAR projects and background information of the facilitating researchers. All three researchers had a positive attitude towards both PAR and the healthy behaviour they wanted to promote. The researchers were highly motivated to co-create with children and adolescents and expected empowerment and effective interventions adjusted to the target population to be outcomes of the PAR. Additional File 1 shows an overview of the composition of each Action Team per project, the frequency and duration of sessions and how the co-researchers were recruited.

**Table 1:** Description of the three PAR projects and background information of the facilitating researchers

Project name (years)	Kids in action (2016–2019) [11, 12]	Healthy sleep project (2017–2020) [13]	LIKE project (2018–2022) [14]
Aim of the project	Improving energy balance-related behaviours in 9–12-year-old children	Promoting healthy sleep in 13–16-year-old adolescents	Improving energy balance-related behaviours in 12–14-year-old adolescents
Background facilitating researcher	M.A. (born in 1990) is a female bicultural Dutch researcher of colour with experience in participatory research, a Bachelor in Human Movement Sciences and a Master in Global Health	A.V. (born in 1991) is a female white Belgian researcher, with experience in youth work, a Bachelor in Nursing and a Master in Health Promotion	H.E. (born in 1992) is a female white Dutch researcher with experience in participatory research, a Bachelor in Health & Life sciences and a Master in Management, Policy-Analysis and Entrepreneurship in Health and Life Sciences
Location of the project	This project took place in an ethnically diverse, lower socio-economic community in the North of Amsterdam, the Netherlands, with a high number of children with overweight or obesity and children growing up in low-income households	This project took place in three secondary schools in Flanders, Belgium. Two schools offered general and technical education to children from families with mainly a higher socio-economic status (SES), and one school offered technical and vocational education to children from families with mainly a lower SES	This project took place in an ethnically diverse community in Amsterdam East, the Netherlands, with a relatively high number of adolescents with overweight or obesity
Medical Ethics Committee	The Medical Ethics Committee of the VU University Medical Center approved the study protocol (2016.366)	The Medical Ethics Committee of the Ghent University approved the study protocol (B670201630466)	The Medical Ethical Committee of the VU University Medical Centre approved the study protocol (2018.234)
Action Teams	In the first 2 years Action Teams were installed at the four public primary schools in the community. In the third year, a Youth Council was started with representatives of the schools. M.A. co-hosted the sessions together with community partners so they would be able to gradually take over the facilitation of the Youth Council (also after Kids in Action ended)	Action Teams were installed at three secondary schools. The project lasted 2 years in the schools offering general and technical education and 3 years in the school offering technical and vocational education	Action Teams were installed at two secondary schools offering lower general education in the selected community. The project lasted 3–4 years in the schools

### Design participatory sessions

Across the three PAR projects, sessions were typically structured with a check-in, the main part of the session and a check-out. The check-in was used for an active game for fun and team spirit and to (re)state the goal of the session and project. During the main part of the session the Action Teams worked on the research topic through varying exercises. We used energizers to help the co-researchers regain their focus or energy when needed. An example was letting the co-researchers play rock-paper-scissors with their whole body for a few minutes. The co-researchers suggested or rejected games or energizers when they liked or disliked a specific game or energizer. Additionally, during some sessions, we applied capacity building to teach the co-researchers certain skills. In two of the PAR projects, another academic researcher, announced as a “research expert” was invited and explained research methods and ethics. In all three projects, the co-researchers acquired organizational skills by being intensively involved and taking the lead throughout the PAR process. During the check-out, we encouraged the co-researchers to summarize the session and plan the next session.

### Reflection form

The leading academic researchers (H.E., A.V., M.A.) from the three PAR projects used the same standardized reflection form to reflect on the group process as well as on their own role as a facilitator. This form was developed using relevant literature on PAR and the facilitation of group sessions as part of the Kids in Action project in 2016 in Amsterdam, the Netherlands [11] and later applied in the Healthy Sleep Project in Ghent, Belgium [13] and the LIKE-project in Amsterdam [14].

The three facilitating researchers (H.E., A.V., M.A.) filled in the reflection form after each PAR session (Table 2). The first part of the reflection form contained 10 items (statements or questions) about the group process, whereas the second part of the reflection form contained 6 items (statements or questions) prompting the facilitator to reflect on their own role as a facilitator. Statements could be answered with −−, −, 0, + or ++, and additional information could be added when necessary. All statements and open questions invited the researcher to reflect on what went well during the session and what could be improved in regard to meeting the principles of participatory research (i.e. “Everyone could give their opinion”) and facilitating collaboration as a team (i.e. “Facilitators had a positive influence on the group atmosphere”).

**Table 2:** Reflection form

Reflection form	
Group process	
The goal of the meeting was clear	(−−, −, 0, +, ++) (optional: additional information)
Everyone participated	(−−, −, 0, +, ++) (optional: additional information)
Everyone could give their opinion	(−−, −, 0, +, ++) (optional: additional information)
The atmosphere/vibe was pleasant	(−−, −, 0, +, ++) (optional: additional information)
Creativity	(−−, −, 0, +, ++) (optional: additional information)
Capacity building: the children learned something new	(−−, −, 0, +, ++) (optional: additional information)
What went well? (group level)	Open question
Which qualities did arise within the group?	Open question
How can we promote the qualities within the group more?	Open question
How can the group process be improved?	Open question
Role of the facilitator	
Facilitators were clear	(−−, −, 0, +, ++) (optional: additional information)
Facilitators involved everyone	(−−, −, 0, +, ++) (optional: additional information)
Facilitators had a positive influence on the group atmosphere	(−−, −, 0, +, ++) (optional: additional information)
What went well (individual level)?	Open question
Which personal qualities arose during the meeting?	Open question
What can be improved according to the facilitator role?	Open question

### Analysis

Data used for analysis included answers to the open questions in the reflection forms as well as the optional additional information provided to the statements. Grading (−−, −, 0, +, ++) of the statements in the reflection forms was not included in the current study. We used thematic analysis by Braun and Clarke to identify, analyze and interpret themes within our qualitative data using the following phases (1) familiarizing of the data, (2) generating initial codes, (3) searching for themes, (4) reviewing themes, (5) defining and naming themes, and 6) producing the report [15]. First, we started with familiarizing ourselves with our data by (re-)reading several reflection forms filled in by various facilitators. Then, we generated initial codes based on core principles of facilitating a PAR session that were found through a pragmatic literature search, including scientific articles, manuals, guides and frameworks on participatory research, co-creation and facilitation [5, 10, 16–24]. We individually searched literature using (scientific) search engines and summarized relevant literature. We discussed the summaries in a face-to-face meeting, and from these we identified guidelines to facilitate the PAR process and clustered the guidelines in themes. Subsequently, we checked whether the defined themes matched the different items of the reflection form, ensuring that the themes would be useful in coding the reflection forms. The defined themes matched most of the items from the reflection form; items that were not represented in the coding book could be added at a later stage while coding.

The three facilitating researchers filled in 252 reflection forms across the three PAR studies (H.E.: 84, A.V.: 69, M.A.: 99). To ensure inclusion of reflection forms on a diversity of PAR sessions, we first sorted the reflection forms by Action Team per school and research phase: (1) the needs assessment phase, (2) the action development phase, and (3) the implementation and evaluation phase, as each Action Team and each phase of the project required a different facilitation approach. For each project, we randomly selected forms from each phase per Action Team until data saturation. Each researcher only coded the reflections forms from the sessions she facilitated, to enable including contextual information and to prevent misinterpretation.

To test and fine-tune the codebook, each researcher searched for themes in three reflection forms from the sessions that she facilitated, using NVivo 11. Then we reviewed the themes, which led to new codes, re-ordering of existing themes and subthemes, and specifying definitions of codes. Afterwards, we individually coded reflection forms from the sessions that we facilitated ourselves (135 in total; H.E.: 41, A.V.: 41, M.A.: 53) using the finalized codebook. To determine whether data saturation was reached, we compared the coded segments. When no additional codes arose during comparison, we concluded that data saturation was reached and no additional reflection forms needed to be included; however, small changes were made in defining and naming the themes in the codebook. For example, similar sub-codes were better defined or merged. Using this final codebook, we checked the coded segments from the reflection forms from our own projects again. Together we discussed the segments of each code and made a summary of the findings per code, which we used to produce the paper.

## Results

Additional File 2 presents an overview of the coding scheme. The coding scheme consisted of 10 themes, that were grouped into 3 subthemes: (1) preparing the PAR sessions – including (i) design of the sessions, (ii) guarding the scope of the project, and (iii) facilitation skills; (2) managing a safe, positive and functional atmosphere during PAR sessions – including (iv) ownership, (v) capacity building, (vi) functional atmosphere, (vii) positive atmosphere and (viii) safe atmosphere; (3) dealing with influencing factors – including (ix) circumstances and (x) group dynamics. The results section is structured according to these three themes. For each theme, detailed information and specific examples are given to illustrate the lessons learned. Finally, each theme ends with a recapitulation of lessons learned from the reflection forms, offering a quick overview of lessons learned.

### Preparing the sessions

Sessions were more successful when prepared in detail, including which participatory methods to use, a time schedule, a “plan B”, and access to a quiet location where the setup could be changed to fit the session and tasks. A successful meeting entailed reaching all the aims for the meeting within the scheduled time and when decision-making was shared with the co-researchers. Starting a session with explaining the goal of the session and how it fitted the overall aim of the co-research helped to keep the co-researchers involved and motivated. When the goal of the session was not clear for the co-researchers, sessions were more chaotic and difficult to facilitate.

*This session wasn’t as well prepared, which shows from the group. It might be a good idea to give a brief overview of what needs to be discussed at the beginning of each session.* – Healthy Sleep Project

*I explained in advance the expectations of this meeting which made it [the aim] clear. Doing so gave me more time to chat and connect with everyone when they were working by themselves.* – LIKE project

When a session was well prepared, facilitators could be more flexible and more easily adapt to the circumstances. Examples are adapting an assignment to fit the atmosphere or using the co-researchers’ alternative ideas for the assignment. Preparing a time schedule consisting of a few topics definitively necessary to discuss in that session and a few optional topics for when time was left allowed for more flexibility. Also preparing alternative plans for unexpected influencing factors (see “Dealing with influencing factors”) enhanced flexibility, for example when the regular meeting room could not be used and the meeting took place in the school hallway. Active games as part of the check-in also helped co-researchers to be more focused during the session. This was similar for the main part of the session, where active and creative participatory methods ensured that co-researchers enjoyed the session and kept their focus. On the other hand, co-researchers sometimes became too energized from an energizer, and had a hard time to refocus.

*Woosh [an energizer] was nice for building a good atmosphere, and as an energizer it enables them to become “energized”. However, I think it was not an ideal exercise for this group, particularly if a more serious task is next.* – Healthy Sleep Project

Before the start of a session, the room in which the session would take place was prepared. When sessions were conducted at school, a more informal atmosphere was created by adjusting the setting to not resemble a school setup. Distracting objects were removed out of the room or furniture moved to improve the efficiency of the session. Tables were, for example, spread across the room so subgroups could work together without getting distracted by others (see “Managing the positive, safe and functional atmosphere during PAR sessions”). The focus of some co-researchers seemed to improve when going outside or being more physical activity during a session.

*Today we were sitting in the sitting area [where children can sit on the floor], which doesn’t work well. On the other hand, we can move around more, compared to sitting at a table, which works better for the more restless individuals.* – Kids in Action.

The main lessons from this theme are: (1) prepare and share the structure of the meeting and start each session with explaining the session goal, (2) plan for flexibility, (3) select various active and creative participatory methods, and (4) play with various meeting areas and setups.

### Managing the safe, positive and functional atmosphere during PAR sessions

When co-researchers had fun and shared in decision-making, this resulted in more successful sessions in which progress could be made. Therefore, ensuring a safe, positive and functional atmosphere appeared important.

Creating a good relationship with the co-researchers contributed to ensure a safe atmosphere. Making agreements or rules together with the co-researchers at the start of or during the PAR process facilitated a safe and functional atmosphere. Co-researchers were encouraged to think of rules themselves regarding creating and maintaining respectful and fruitful relationships within the group. Examples of agreements included listening to each other or taking turns to talk. The facilitators and co-researchers referred to these agreements when necessary. When difficult situations arose that were not covered by these agreements, the facilitator had to indicate boundaries and be strict to avoid chaos. This did not necessarily create a negative atmosphere: the clarity and structure created by the boundaries actually improved the atmosphere in most cases. Only in some instances was it necessary to address the co-researcher(s) who distorted the session separately after the session. In the case where a co-researcher’s participation had to be ended, this decision was discussed together with the co-researcher, their parents and the school teacher.

*[group was misbehaving] You want the working environment to remain healthy, in order to support appropriate behaviour. For instance, to enable this, I established my boundaries, yet remained pleasant in this situation.* – Healthy Sleep Project

Furthermore, to ensure a safe atmosphere, one of the most important tasks of facilitators was to make sure that each co-researcher felt acknowledged and heard during the session. In situations where a dominant person took over the conversation and prevented others from expressing themselves, it helped to ask if everyone agreed with what was being said and to address individuals. This sometimes opened unexpected conversations or perspectives. A useful approach was to let all co-researchers write down their opinion and then let everyone share what they had written down, so everyone’s opinion was considered. Being hasty as a facilitator could be a pitfall, as then only the loudest voices were heard and there was less time to show appreciation of other co-researchers. Expressing appreciation to all co-researchers appeared important, as it made them feel acknowledged and it increased their motivation.

Taking time to have fun with the group and getting to know each other rather than being strict to increase efficiency contributed to a positive atmosphere. Playing a game or having an informal conversation sometimes led to deviations from the plan, yet it resulted in a stronger relationship with the co-researchers. Co-researchers often had personal questions for the facilitator; taking the time to answer such questions promoted having an open and strong relationship with the co-researchers, which aided a safe, positive and functional atmosphere. This actually allowed us to be more strict when necessary. Creating a positive atmosphere was sometimes at the expense of a functional atmosphere, but a good team spirit increased the motivation and, thus, the efficiency in the longer term.

*I share a lot about myself, am able to laugh along as well as join conversations that others initiate (it really interests me too). It makes for a pleasant atmosphere, but perhaps this is less favourable for focusing purposes.* – Healthy Sleep Project

*We sometimes had to reprimand the others, but maybe we should have been more stern to maintain a better working environment.* – Kids in Action

*It would be nice if they [the adolescents] could listen to each other better without me needing to raise my voice. Therefore, we can perhaps still be more strict or ask them how we can manage that together (refer to the rules?)* – LIKE project

Several approaches to moderate the ongoing conversation and guide co-researchers during their thought process stimulated a functional atmosphere in which shared decisions could be made and ideas could be elaborated on. Examples are: asking the co-researchers questions, offering them new angles or summarizing what they had said. It worked even better to ask a co-researcher to summarize what had been said. Co-researchers were also encouraged to think creatively and think of alternatives when an idea appeared not feasible. This was sometimes considered difficult by facilitators because they did not want to immediately destruct an idea and demotivate the co-researchers. This mainly happened in groups with younger co-researchers, who had many ideas but struggled with feasibility, especially in the beginning of their participation. In some groups, especially with co-researchers following vocational education, it was necessary for the facilitators to bring in some ideas as inspiration to facilitate the brainstorm. Co-researchers could then explain why they did or did not like the idea and build on from there.

*The process was very smooth and natural. I was able to ask the right questions and leave silences to allow more ideas to surface. I had a good attitude to brainstorming. (We also get training for that at [name organization]).* – Healthy Sleep Project

*I think that, together, we were well engaged with brainstorming. Actually, some of those involved in this had come up with the majority of ideas. In some instances, ideas necessitated adaption, but I do think almost all the ideas came from the children.* – Kids in Action

When concrete tasks had to be worked out, it was more functional to split the action group into smaller groups to work on a separate task. Everyone could then contribute more actively, co-researchers worked more focused and personal strengths arose. It was useful to have one facilitator per subgroup if possible, to let subgroups work at different tables or in different rooms, and to think about the subgroup composition. Co-researchers could choose a task or subgroup themselves or were divided by us according to age, sex, strengths and so on. To keep the co-researchers focused, it helped to use visual support during explanations or group discussions, for example, writing or drawing what co-researchers talked about on a whiteboard or flip-over. At the end of the session, subgroups reported to each other what they had worked on using these visual summaries, so that every co-researcher stayed up to date about the progress and they could decide as a group on the next steps. Staying up to date about all decisions in the project was important for the sense of ownership among co-researchers. Therefore, it was also important to inform co-researchers about meetings that took place without them (e.g. between facilitators and school board or advisors) and ask the opinion of co-researchers about decisions that had to be made in response to such meetings.

Finally, encouraging co-researchers to take the lead – by asking them what they wanted to do themselves and giving them as many responsibilities as possible – increased their sense of ownership. This was aided by the time spent on capacity building. Co-researchers were very capable of gaining research and organizational skills. At the end of the session, co-researchers were often asked what they would like to do the following session, which helped to keep them engaged and design sessions that fit their interests. This again had a positive influence on their sense of ownership. When the sense of ownership in co-researchers increased, co-researchers started to be more assertive.

*The children cooperated on their own initiative to set up a questionnaire.* – Kids in Action

*I was good at relinquishing control and letting [one of the adolescents] be more in charge. This allowed them to gain some leadership. Nonetheless, I was able to successfully take the lead again when needed.* – LIKE project

*The adolescents often asked each other for advice, engaged in dialogue and came up with solutions for the others. This made it a super interactive session.* – Healthy Sleep Project

The main lessons that can be concluded from this theme are: (1) make good agreements or rules together about how to create and maintain a respectful and fruitful relationship within the group, (2) take time to have fun and to get to know each other, (3) guide co-researchers in structuring their ideas, (4) make sure everyone is heard, (5) split the group into smaller subgroups for concrete tasks, (6) be transparent about meetings that take place without the co-researchers being present, and (7) encourage co-researchers to take the lead.

### Dealing with influencing factors

The characteristics, moods and emotions of facilitators and the co-researchers often influenced the session and all attendees. For example, when facilitators lacked energy, this reflected on the co-researchers. In addition, co-researchers could, for example, be nervous for exams that were coming up, excited about a good grade or on edge because of bad weather conditions. Those factors were unpredictable and had to be dealt with on the spot. Other factors, such as co-researchers’ personality, were more predictable and, therefore, methods could already be adapted to this.

*You can clearly notice your own influence on the group. Having been so busy lately, I felt like chilling with them and not stressing much about time (or people not paying attention). Today I enjoyed that (instead of the strict time management and progress) = > outcome: they enjoyed themselves a lot and were outgoing BUT their input was of lower quality than last week.* – Healthy Sleep Project

To a certain extent, facilitators tried to neutralize the effect of hindering factors. If facilitators were, for example, in a hurry and felt stressed, it helped to take 10 min before the session to relax. If that did not work and an extra facilitator was present, the second facilitator was given more responsibility. If co-researchers were distracted, it helped to let them share what was on their mind instead of urging them to immediately start working on the project. In this sense, the dynamics and energy of the group had to be considered constantly. Dealing with these varying circumstances required different facilitation skills and methods from the facilitators. For this, it helped to have taken facilitation courses or joined more experienced PAR researchers earlier on which enabled learning by doing.

*Z. came in later today. He wanted to join the conversation right away, but he did not really know what we were talking about. I should have let the two other girls get him up to speed first.* – Kids in Action

The dynamics in the group were also influenced by the composition of the group. The reflection forms indicated that when there were both dominant and shy co-researchers in one group, the moderating role of the facilitator became more important. With younger co-researchers, more pedagogical and facilitation skills were needed, as they could be more energetic and lost their focus more quickly. When a co-researcher impeded the atmosphere or bullied others, skills to manage tough situations were needed, such as having a serious one-on-one conversation with that co-researcher.

The PAR process ran more smoothly when all co-researchers were on time and attended all sessions, as it was very distracting when co-researchers showed up late and missed out what had already been discussed. It was useful to decide on the frequency and time of sessions together with the co-researchers, as some preferred to have sessions in the morning (but others were still half-asleep then), while others preferred the late afternoon (but others were half-asleep by then).

*I could direct them to attend weekly. Additionally, the kids attending every week are much more involved and their tasks are more evident. For the ones only in occasional attendance, I think the work is less fulfilling since they don't have a clear understanding of what their purpose is.*—Kids in Action

The main lessons that can be concluded within this theme are: (1) the facilitator’s mood and energy is reflected in the co-researchers, (2) a facilitator needs a diverse skillset (e.g. to adequately handle different personalities within the group), and (3) to decide on a meeting time and frequency together with the co-researchers.

## Discussion

This study aimed to illustrate how a facilitator can contribute to a successful PAR process based on a standardized reflection form from three PAR projects with children and adolescents. Several lessons learned that are likely also valuable for PAR with adult co-researchers. The added value of our study over existing more general guidelines [5] is that our findings result from in-depth qualitative analysis of a standardized reflection form used in three separate projects. Our reflection form proved to be a valuable tool for uncovering detailed examples and specific recommendations for the PAR process and our role as facilitator, enabling facilitators to improve their facilitation during the participatory process. These findings provide researchers who want to conduct PAR with a more practical summary of best practices for facilitators and detailed examples. The insights provided regarding ethics, methods and evaluation may be informative for future PAR projects and researchers wanting to develop or further elaborate on a co-creation methodology.

Successful sessions need considerable preparation. Facilitators need to maintain a safe, positive and functional atmosphere during sessions which can be influenced by the mood of both the facilitator and co-researchers. These findings confirm previous research [22–24].

Engaging with co-researchers on a personal level and ensuring a good connection with the group seemed important, confirming findings by facilitators from other PAR projects [25]. This contributed to a trustworthy relationship where all co-researchers felt they could speak their mind and be themselves, which in turn positively influenced the sense of ownership and control over the process, which are core principles of PAR [5]. Co-researchers took a more leading role in the sessions when they felt more ownership. These observations correspond with the Theoretical Framework of Symbolic Interactionism, which suggests that individuals’ engagement is based on their personal meaning in life, which is shaped by their interactions with others [26]. However, facilitators should be aware that closer relationships with co-researchers also bring greater potential for exploitation [27]. Since participation is often voluntary, it is critical that decisions are made together with the co-researchers, for example, about the aim of the study, their preferred role and how much time they want to invest.

Above all, our findings show that a flexible attitude from the facilitator in being goal oriented, being able to build strong relationships with co-researchers and having strong facilitation skills are essential for a successful PAR process. Especially finding a balance between moderating the conversation and letting co-researchers steer the conversation (in sometimes directions unrelated to the research topic) is a common challenge for PAR facilitators [28–30]. Sharing power with adolescents might be challenging for facilitators, and the most recurrent criticism on Youth PAR is the risk that researchers keep too much control. This can result in tokenism and falsely claiming collaboration with adolescents [31]. An important responsibility lies with the facilitator and their integrity regarding this challenge: facilitators should be compassionate, courageous, honest, humble and righteous and have the ability to have moral insight about a situation to establish ethical relationships in participatory research [32]. As facilitators play an important role in the PAR process, critical reflection through journaling or group dialogues on their behaviour and thoughts during collaboration with co-researchers is recommended [33]. Continuous reflection throughout the PAR process will provide insights into the facilitators’ influence on the research process, the social dynamics with co-researchers and power differentials that arise [8, 33]. As a helpful tool to guide this reflection, we provide the adapted reflection form in Additional File 3.

Based on the results of this study, we adjusted the reflection form for future use (Additional File 3). As a functional atmosphere and suitable working methods emerged as important themes, we added the following questions to the reflection form: “Were all goals of the session reached? If not, why not? If yes, how were they reached?” and “How were the used participatory methods received and did they give the anticipated output?”. As reflecting on the influence of the facilitators’ mood and personal characteristics on the session was not part of the reflection form, we added the following question: “How did the mood and personal characteristics of the facilitator influence the session?”. Finally, as equal collaboration and shared decision-making must be pursued at all times during PAR [6], we added the following questions: “To what extent were co-researchers in the lead during the session? How did this become visible? How was this reached?”.

Some limitations of the study need to be acknowledged. This study was conceived after the data had been collected, and we did not know in advance that we would combine the reflection forms of the three projects during the PAR process. Therefore, not all situations were described elaborately or easily interpretable for others. Furthermore, as the grading system within the reflection form (−−, −, 0, +, ++) was not concretized, we interpreted this differently when filling out the forms and, therefore, could not include the grading in the current study. We changed the grading system to disagree, neutral and agree in the updated reflection form in Additional File 3. Another limitation of our study is the potential bias introduced by facilitators analysing their own reflection forms, which was necessary to include contextual information and to prevent misinterpretation. Although we believe that our findings are of use to a wide spread of PAR researchers, researchers using our recommendations should take the context in which these originated (PAR with children and adolescents in West Europe) into account.

A strength of this study was that a large amount of data was collected over long time periods (3–4 years for each of the projects). Another strength is that the three PAR projects that used the reflection forms worked with co-researchers from different age groups, social backgrounds and educational levels. This led to insights that can be used for a wider range of PAR projects. A third and final strength of this study is that real-world data were analysed on the basis of already identified principles of facilitating PAR processes, linking available core principles of facilitating PAR processes in literature to data.

## Conclusion

In-depth qualitative analyses of a standardized reflection form used in three different PAR projects resulted in various lessons to support facilitators in collaborating with co-researchers in PAR projects: 1) when preparing PAR sessions, facilitators should plan for flexibility, include active and creative participatory methods, play with varying locations and set-ups and let co-researchers influence the pace and clearly state the goal at the beginning of the sessions; 2) to ensure a safe, positive and functional atmosphere, make good agreements on how to work together and make sure everyone is heard as well as take time to have fun and get to know each other; 3) to ensure ownership and shared decision-making, facilitators need to encourage co-researchers to take the lead and be transparent about additional meetings that facilitators or involved researchers have without the co-researchers; 4) to handle influencing factors such as the mood of co-researchers, a facilitator needs a diverse skillset.

### Supplementary Information

Additional file 1

Additional file 2

Additional file 3

## Data Availability

The reflection form and final codebook are shared in the study and Additional file. The filled in reflection forms cannot be shared because individual privacy could be compromised.

## Abbreviations

PAR: Participatory Action Research
SES: Socio-Economic Status
