# Supplementary material for: Facilitating co-research: lessons learned from reflection forms within three participatory action research projects
Source: Health Res Policy Syst. 2024 Aug 23;22:117. doi: 10.1186/s12961-024-01210-x (PMC11342652; doi:10.1186/s12961-024-01210-x)
Supplement: Supplementary file 1 — Additional file 1 [file 12961_2024_1210_MOESM1_ESM.docx]

**Additional file 1. Overview of the composition of each Action Team**

Table 1: composition of Action Teams in the Kids in Action project

|  | **Year 1 (2016-2017)** | **Year 2 (2017-2018)** | **Year 3 (2018-2019)** |
| --- | --- | --- | --- |
| **School 1** | Seven children (6 girls), weekly sessions after school hours of 45 minutes, followed by 45 minutes of sports. Children were part of their school’s student board. | Eight children (7 girls, 2 from previous year), weekly sessions after school hours of 45 minutes, followed by 45 minutes of sports. One of the new children was chosen by a teacher, two were chosen by the two children from the previous year, the other children asked if they could join on their own initiative. | Five representatives (2 girls). All children from grades 6-8 could sign up. |
| **School 2** | Six children (2 girls), every two weeks a session of one hour during school hours. Teachers chose the children. | 6 children (2 girls, 4 boys; 4 from previous year), every 2 weeks a session of 1 hour during school hours. The two new children were chosen by the teachers. | No representatives as no children signed up |
| **School 3** | Six children (1 girl), weekly sessions after school hours of 45 minutes, followed by 45 minutes of sports. All children from grades 6-8 could sign up. | Six children (2 girls, 4 boys; all new), weekly sessions of 45 minutes, followed by 45 minutes of sports. All children could sign up. | Three representatives (1 girl). All children from grades 6-8 could sign up. |
| **School 4** | Six children (3 girls), every two weeks a session of one hour during school hours. Physical education teacher chose the children. | School did not participate. | Five representatives (1 girl). All children from grades 6-8 could sign up. |

Table 2: composition of action teams in the healthy sleep project

|  | **Year 1 (2017-2018)** | **Year 2 (2018-2019)** | **Year 3 (2019-2020)** |
| --- | --- | --- | --- |
| **School 1** | Ten adolescents (3 girls; 7 from the second grade of secondary school), weekly sessions during 1 hour lunch break. All adolescents from the second and third year could sign up voluntarily. | Nine adolescents (3 girls; 6 from the third grade), weekly sessions during 1 hour lunch break. Same participants as in the Action Team in Year 1. | Project was completed in Year 2. |
| **School 2** | Seven adolescents (5 girls; 6 from the second grade), weekly sessions during 1 hour lunch break. All adolescents from the second and third grade could sign up voluntarily. | Seven adolescents (4 girls, 4 from the third grade), weekly sessions during 1 hour lunch break. Three participants of the Action Team were the same as in Year 1, new participants were friends of participants or recruited by a new call to sign up voluntarily. | Project was completed in Year 2. |
| **School 3** | Twelve adolescents (no girls, 1 from the second grade), weekly sessions during 1 hour lunch break. All adolescents from the second and third year could sign up voluntarily. | Twelve adolescents (no girls, 8 from the third grade), weekly sessions during two months during teaching hours of a technical education class. Two teachers were willing to let the sessions take place within their teaching hours, all adolescents part of the class participated. | Twelve adolescents (no girls, 7 from the third grade), five sessions during teaching hours of a technical education class. Two teachers were willing to let the sessions take place within their teaching hours, all adolescents part of the class participated. |

Table 3: composition of action teams in LIKE

|  | **Year 1 (2018-2019)** | **Year 2 (2019-2020)** | **Year 3 (2020-2021)** | **Year 4 (2021-2022)** |
| --- | --- | --- | --- | --- |
| **School 1** | Seven adolescents (5 girls), every week a session of 45 minutes during school hours. All adolescents in the first two grades of secondary school could sign up. | Six adolescents (1 girl), every week a session of 45 minutes during school hours. All adolescents in the first two grades of secondary school could sign up. | Four adolescents (4 girls), every week a session of 45 minutes during school hours. All adolescents in the first two grades of secondary school could sign up. | Due to Covid-19 the implementation of an action was postponed to year 4. Two girls from year 3 continued their participation in seven sessions. |
| **School 2** | Six adolescents (1 girl), every week a session of 2 hours during school hours. All adolescents in the first two grades of secondary school could sign up. | Five adolescents (5 boys), every week a session of 2 hours during school hours. All adolescents in the first two grades of secondary school could sign up. | Eight adolescents (2 girls) in period 1, four adolescents (1 girl) in period 2 and 3. Every week a session of 2 hours during school hours. All adolescents in the first two grades of secondary school could sign up. | Project was completed in Year 2. |

^* From the LIKE-project only data from the secondary schools (12-14 year old adolescents) are included in the current study because HE facilitated these sessions.^
