# Supplementary material for: Facilitating co-research: lessons learned from reflection forms within three participatory action research projects
Source: Health Res Policy Syst. 2024 Aug 23;22:117. doi: 10.1186/s12961-024-01210-x (PMC11342652; doi:10.1186/s12961-024-01210-x)
Supplement: Supplementary file 2 — Additional file 2 [file 12961_2024_1210_MOESM2_ESM.docx]

**Additional file 2. Translated codebook**

| Name | Description |
| --- | --- |
| Capacity building | Co-researchers gain skills. |
| Research skills | Co-researchers learn skills to conduct their own research, e.g. what are research ethics, how to formulate a research question, what types of research methods are there |
| Organizational skills | Co-researchers learn skills to find a step by step solution, e.g. what is needed to develop and implement an action, what things should you think about |
| Functional atmosphere | Create an atmosphere that ensures that work is done efficiently and that the goal of the session is achieved |
| Making agreements | Make rules for a guaranteed functional atmosphere |
| Guided thinking | Guiding co-researchers in the thinking process, asking the right questions to reach concrete results, without being dominant |
| Check in | Start of the session, welcome, games |
| Check out | Close the session, recap, plans for next session |
| Encourage co-researchers to participate | Actively involving the co-researchers (e.g. by asking questions, but also choosing an appropriate method that challenges them to participate) and ensuring that they participate |
| Energy | Get the energy at the right level to achieve a functional atmosphere, e.g. with energizers |
| Focus/concentration | Trying to improve focus/concentration or lack of focus |
| Group work | Divide co-researchers into subgroups |
| - Group composition | How best to divide subgroups |
| - Dividing assignments | What kind of assignments do you give the subgroups, are the assignments the same or different |
| - Overview supervisor | Does the supervisor still have an overview of the subgroups or not? |
| - Collaboration | Co-researchers work better together when they work in subgroups |
| - Participation | Co-researchers participate more actively because they have more to do when they work in subgroups |
| - Individual guidance | More individual guidance (with sufficient supervisors) |
| - Qualities | Qualities of co-researchers stand out because they get to speak more and can do more. Can be coded together with 'can/dare to express' |
| - Focus | Co-researchers have more focus because, for example, there is less distraction, they have a more individual task. Can be coded together with 'focus/concentration' |
| - Efficiency | You get more done when co-researchers work in subgroups |
| Inspiration outcome | Give ideas for concrete actions |
| Moderating | Bring conversation back to topic with concrete questions |
| Summarizing | Summarizing everyone's ideas |
| Group dynamic | Dynamics within the group, how is everyone treated, how are the dynamics influenced by the presence or absence of (one of) the facilitators |
| Changes in the group | The dynamics change because someone is absent or joins |
| Influence of group size | The dynamics change because the group size is different than normal |
| Influence of dominant person | The dynamics are influenced by a more dominant/leader type co-researcher within the group. That co-researcher has a lot of influence on the rest of the group and the dynamics. |
| Disagreement/agitation | The dynamics within the group are restless and there is disagreement |
| Dividing roles | Distribution of roles within the group and how this affects the dynamics |
| - By the facilitator | The division of roles within the group is done by the facilitator |
| - By the co-researchers | The division of roles within the group is mutually arranged by the co-researchers |
| Circumstances | Spatial and other factors the facilitator has to deal with |
| Mood | Mood of the co-researchers, whether or not influenced by previous situations |
| Set-up | Physical set up of the room, e.g. placement of tables and chairs |
| Preparing the room | Prepare the room to fit the meeting and assignments |
| Tranquility | Whether or not the environment is quiet or are there distractions |
| Time | At what time does the session take place and what is the result |
| Design of the session | Preparation, content, structure of the sessions |
| Consistency across the meetings | A common thread/coherence/relationship between the meetings |
| Setting the goal clearly and repeating it | Clarify the purpose of the session and of the project |
| - Clarity co-researchers | Co-researchers know where they stand because the goal is clearly set |
| - Framing the assignment | Clarifying the purpose of the assignment given |
| - Confusion co-researchers | It is noticeable that it is unclear to co-researchers what the intention is because the facilitator did not clearly state the goal (at the beginning of the session) |
| - Positive effect on session | Making clear frameworks/goals at the start of the session has a positive effect on the course of the session |
| Structure | Provide more structure in the session |
| Method | Which way of working/method did the facilitator apply and with what purpose/consequence |
| Active | Co-researchers are physically moving |
| Creative | Innovative method or artistic |
| Fun | Method is experienced as fun |
| Appropriate | Method is suitable for preference/characteristics of co-researchers |
| Division of roles multiple facilitators | How are the roles divided between multiple facilitators or is there a lack of multiple facilitators |
| - Efficiency | Facilitators get more done when there are multiple facilitators present and can give more individual guidance. Facilitators can also manage co-researchers better (links to 'efficiency' in group work) |
| - Division of tasks | Before the session agree together on the (different) division of tasks, it will not go well if you do not do that (e.g who is the observer or lead facilitator) |
| - Who is the other facilitator | Teacher/student/trainee, what can you expect from that person |
| Do boring things yourself | Facilitator does things that are less interesting to co-researchers, or things that are too difficult |
| To prepare | Be prepared for different scenarios |
| Time management | How to organize time throughout the session, how to organize time in advance |
| Ownership |  |
| Encourage control over process | Facilitator encourages co-researchers to take more responsibility |
| Reflection | Reflection occurs during the session between co-researchers and facilitator that leads to greater influence on the process and ownership |
| Division of roles between facilitator and co-researchers | Division of roles, discussing who can and wants to do what, co-researchers who spontaneously take up roles |
| Self-regulation | Co-researchers take matters into their own hands, correct/help each other |
| PAR general | Subcodes are more general, not session specific |
| Positionality facilitator | How does the facilitator relate to the group; age, education level, ethnicity, gender; reflection on the facilitator’s position and insights influence the group and the process |
| Transparency | Be honest in goals, roles and possibilities, what happens to their input, what is their role as a group |
| Positive group atmosphere | Atmosphere in the group, team events, team building |
| Fun | Having fun during the session |
| Team spirit | Things the facilitator does to encourage team spirit |
| Informal contact | Room for distraction, fun |
| Games | Using games during the session to have fun |
| Showing appreciation | Positive reinforcement, saying they are doing well, to create a positive atmosphere, motivating, encouraging ownership |
| Guarding the scope of the project | Making it clear when something falls outside the scope of the project. |
| Feasibility | Keeping ideas realistic |
| Skills facilitator | skills or qualities of the facilitator |
| Communication skills | Knowing how to communicate in certain circumstances, adjusting your language level, conveying your thoughts and ideas. Non-verbal communication could also be included. |
| Handing over control | Sharing power, letting go, handing over control to children |
| Being energetic | The facilitator is energetic herself |
| Facilitation style | The spectrum of non-authoritarian to authoritarian, which the facilitator adjusts to the group. Being or not being on the same level as the group. |
| Flexibility | The facilitator adapts to the circumstances, deviates from preparation, easily responds to what happens during a session |
| Room for development | Give children the space to work out ideas or to develop their own competences |
| Managing difficult situations | How does the facilitator handle difficult situations in the group or with co-researchers? |
| Pedagogical skills | knowledge about how to deal with the co-researchers in certain situations during the session |
| - Expectations of the facilitator towards co-researchers | Knowing what to expect from certain co-researchers and adapting your methods accordingly |
| - Indicate boundaries | Being strict, indicating when something is not possible |
| Planning skills | Skills regarding planning of the session. |
| Social skills | Assessing relationships with others, empathy, making someone feel comfortable, relational, dealing with others |
| Safe atmosphere | An open atmosphere in which everyone can say what he or she wants and is accepted for who he or she is. |
| Making agreements | Making agreements in order to guarantee a safe atmosphere |
| Feeling free to express yourself | Co-researchers feel free to safely express themselves |
| Recognizing each child | Listen to everyone’s input and showing this. |
| Being able to express yourself | There is room to express yourself |
