# Supplementary material for: Facilitating co-research: lessons learned from reflection forms within three participatory action research projects
Source: Health Res Policy Syst. 2024 Aug 23;22:117. doi: 10.1186/s12961-024-01210-x (PMC11342652; doi:10.1186/s12961-024-01210-x)
Supplement: Supplementary file 3 — Additional file 3 [file 12961_2024_1210_MOESM3_ESM.docx]

**Additional file 3. Improved reflection form based on the results of this study**

| **Reflection**  -disagree, 0 neutral, + agree* (- = you don’t agree with the statement, 0 = you neither agree or disagree with the statement or can’t say anything about it, + = you agree with the statement) | |
| --- | --- |
| **Group process** | |
| The goal of the meeting was clear | (-,0,+) (optional: additional information) |
| Everyone participated | (-,0,+) (optional: additional information) |
| Everyone could give their opinion | (-,0,+) (optional: additional information) |
| The atmosphere/vibe was pleasant | (-,0,+) (optional: additional information) |
| The co-researchers could show their creativity | (-,0,+) (optional: additional information) |
| Capacity building: the children learned something new | (-,0,+) (optional: additional information) |
| What went well? (group level) | Open question |
| How were the used participatory methods received and did they give the anticipated output?* | Open question |
| Which qualities did arise within the group? | Open question |
| How can we promote the qualities within the group more? | Open question |
| How can the group process be improved? | Open question |
| Were all goals of the session reached? If not, why not? If yes, how were they reached?* | Open question |
| To what extent were co-researchers in the lead during the session? How did you this become visible? How was this caused?* | Open question |
| **Role of the facilitator** | |
| Facilitators were clear | (-,0,+) (optional: additional information) |
| Facilitators involved everyone | (-,0,+) (optional: additional information) |
| Facilitators had a positive influence on the group atmosphere | (-,0,+) (optional: additional information) |
| What went well (individual level)? | Open question |
| Which personal qualities arose during the meeting? | Open question |
| How did the mood and personal characteristics of the facilitator influence the session?* | Open question |
| What can be improved according to the facilitator role? | Open question |

*Questions indicated with an asterisk were added based on the results of this study
